# Pacemaker Lead Migration and Ventricular Perforation in a Patient Presenting with Chest Pain

**DOI:** 10.5811/cpcem.2021.7.52689

**Published:** 2021-09-09

**Authors:** Maria C. Cañizares-Otero, Mauricio Danckers

**Affiliations:** Aventura Hospital & Medical Center, Department of Critical Care Medicine, Aventura, Florida

**Keywords:** pacemaker, ventricular lead, perforation, chest pain

## Abstract

**Case Presentation:**

We describe a middle-age male with a past medical history of second-degree atrioventricular block type II status post permanent pacemaker placement the day prior who presented to the emergency department complaining of chest pain. Electrocardiography showed a non-paced ventricular rhythm. Chest radiograph showed the ventricular pacemaker lead located distally overlying the right ventricle apical area. On further investigation, chest computed tomography showed a perforation of the ventricular wall by the pacemaker lead prompting urgent intervention by the cardiothoracic surgery team for lead replacement and right ventricular repair.

**Discussion:**

Our case illustrates the importance of timely recognition of a perforated pacemaker lead in a patient presenting with chest pain after device implantation. We additionally describe the risk factors for ventricular perforation, initial clinical presentation, and management approach.

## CASE PRESENTATION

**Image 1:**
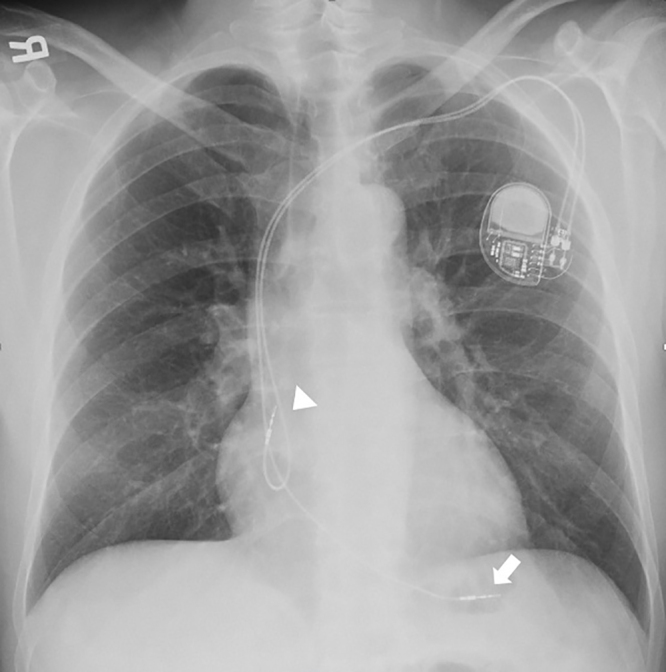
Chest radiograph revealing ventricular pacer lead located distally overlying the right ventricle apical area (arrow) and atrial pacer lead within right atrium area (arrowhead).

**Image 2:**
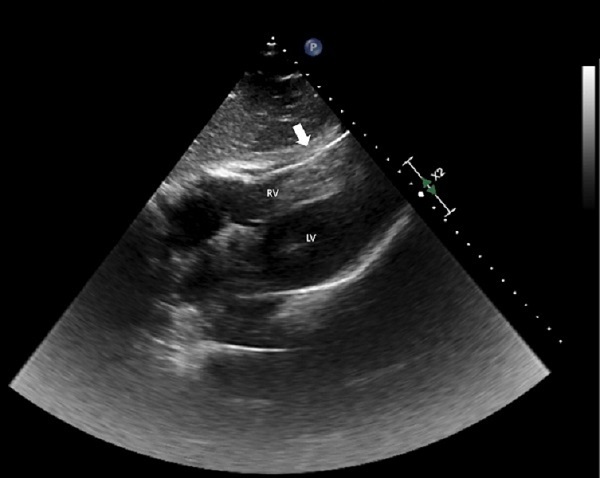
Point-of-care echocardiogram subcostal view during systole showing ventricular pacer lead (arrow) beyond right ventricular chamber. No pericardial effusion is seen. RV, right ventricle; LV, left ventricle.

**Image 3:**
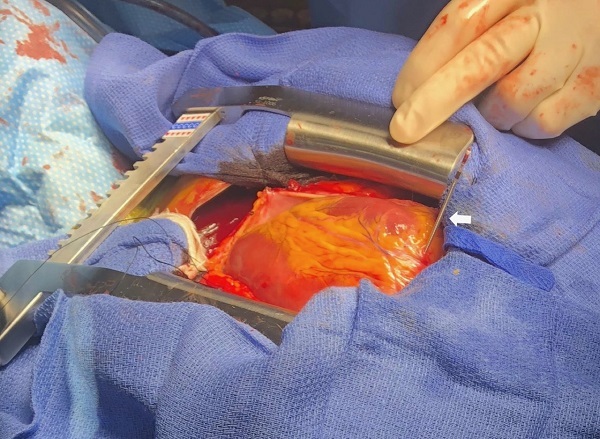
Mediastinal exploration via sternotomy revealing ventricular lead piercing through the right ventricular apex (arrow).

A 52-year-old man with second-degree type II atrioventricular (AV) block and recent dual-chamber pacemaker placement presented to the emergency department with left sided chest pain for the prior two hours. Electrocardiogram showed a second-degree AV block with a ventricular rate of 45 beats per minute. Pacemaker interrogation revealed atrial sensing without ventricular capture. His chest pain was exacerbated with electrical stimulation despite lack of capture and resolved after disabling the pacemaker. Further imaging (chest radiograph [CXR] and point-of-care echocardiogram) revealed ventricular pacer lead mispositioned and a possible cause of acute chest pain (Images 1, 2). Coronal computed tomography (CT) of the chest confirmed lead migration beyond the right ventricle chamber. The patient was emergently taken to the operating room. Exploratory sternotomy confirmed ventricle perforation (Image 3). The patient underwent lead extraction, right ventriculorrhaphy, and epicardial right ventricle pacemaker lead placement. He was discharged home on hospital day four.

## DISCUSSION

Pacemaker lead ventricular perforation is a rare event that occurs in 0.4–5.2% of pacemaker lead placements.^1^ Prompt diagnosis requires high clinical suspicion in patients with chest pain and recent history of device placement or lead exchange. Presentation can be acute, subacute, or delayed; perforation most commonly occurs within the first month of placement.^1^,^2^ Risk factors for perforation include prior temporary pacemaker placement, active fixation leads, female gender, and recent steroid use. The pathophysiology of perforation is attributed to continuous pressure of the thin lead per unit of the myocardial wall.^2^,^3^ Perforation of the right ventricular apex is the area most commonly prone to perforation due to weakness of the wall.

**CPC-EM Capsule**

What do we already know about this clinical entity?

*Pacemaker lead ventricular perforation is a rare complication of pacemaker placement and can present with chest pain, dyspnea, dizziness, syncope, or pacemaker failure.*

What is the major impact of the image(s)?

*Chest radiography followed by point of care echocardiography or computed tomography of the chest are readily available imaging studies to support the diagnosis.*

How might this improve emergency medicine practice?

*Emergency physicians should have a high index of suspicion for pacemaker lead perforation in the clinical setting of chest pain and recent pacemaker placement.*

Chest pain, dyspnea, dizziness, syncope, and pacing or sensing pacemaker failure are commonly encountered clinical scenarios. Point-of-care echocardiogram allows for the clinician to promptly rule out emergent conditions such as hemorrhagic pericardial tamponade. Additional workup includes CXR and echocardiogram to evaluate for pericardial effusion; however, a non-contrast CT is usually required to confirm the diagnosis. Management is targeted based on patient’s clinical stability and the extent of injuries to nearby structures.^4^,^5^ Clinically unstable or symptomatic patients often need emergent surgical repair.
